# Editorial: Mobilome manipulation: engineering microbiomes to counteract antimicrobial resistance

**DOI:** 10.3389/fmicb.2026.1938991

**Published:** 2026-08-07

**Authors:** Govindan Rajamohan, Peter Mullany

**Affiliations:** 1 Council of Scientific & Industrial Research-Institute of Microbial Technology, Chandigarh, India; 2 University College London, London, United Kingdom

**Keywords:** antimicrobial resistance, horizontal gene transfer, mobile genetic elements, plasmidome, resistance genes

Antimicrobial resistance (AMR) is a global problem that compromises antimicrobial treatments and poses significant threats to public health. The rapid spread, emergence, and re-emergence of antimicrobial-resistant bacterial pathogens leading to resistant infections are largely due to transmissible mobile genetic elements (MGEs), the whole Research Topic of which in any particular ecological niche is referred to as the mobilome (Antimicrobial Resistance Collaborators, 2022; Frost et al., 2005; Robinson et al., 2016). Clinically relevant antibiotic-resistant genes are present on a range of MGEs, plasmids, transposons, integrons, insertion sequences, genomic islands, integrative conjugative elements, and bacteriophages that transmit these genes across bacterial species through horizontal gene transfer (HGT) (Hall et al., 2017; Partridge et al., 2018). Therefore, to limit or reverse the spread of MGEs, it is vital to understand the structure and dynamics of the mobilome across diverse environmental niches to develop innovative strategies that preserve the efficacy of existing antimicrobial therapies. This will help in developing strategies to interrupt the transmission pathways by precise inhibition of ARG-carrying pathogens and maintaining the beneficial commensal pool (Bikard and Barrangou, 2017).

The focus of this Research Topic “*Mobilome manipulation: engineering microbiomes to counteract antimicrobial resistance*” was to highlight the research and reviews that will advance our understanding in the emerging area of mobilome biology and its role in dissemination across One Health interfaces. The articles published in this Research Topic advance understanding of the reservoirs of MGEs, the mechanisms that drive HGT, the diversity of ARG dissemination plasmidome, and potential intervention strategies to disturb resistance transmission. The Research Topic represents AMR in distinct microbial and environmental settings, and its contribution to the central theme of uncovering how the mobilome shapes the dissemination of ARGs across bacterial pathogens.

A prominent theme emerging from this Research Topic is the importance of environmental reservoirs of MGEs and the role of plasmids in the dissemination of AMR. Environmental microbiomes represent a potential hotspot/hub for the exchange and movement of ARGs across ecological boundaries.

Luo et al. demonstrated that community air and wastewater environments represent significant yet underappreciated reservoirs of mobile plasmids harboring ARGs. Furthermore, the study demonstrated that transferable ARGs contained on plasmids persist and transmit in non-clinical environments, emphasizing the importance of environmental surveillance in understanding AMR transmission pathways. These findings underscore the urgent need for surveillance beyond healthcare settings and strengthen the concept that environmental mobilomes constitute a global resistome and play a pivotal role in the emergence and dissemination of ARGs.

Further complementing this ecological perspective, de Souza et al. conducted a large-scale plasmidome and carbapenem mobilome analysis involving thousands of carbapenemase-encoding plasmids. Comparative analysis of plasmids revealed complex mobilization networks encompassing insertion sequences, transposons, and integrons associated with clinically relevant resistance gene cassettes. Furthermore, their study identified complementary mobilization strategies mediated by structurally specialized integrons, transposons, and insertion sequences in the transmission of carbapenem resistance. Importantly, the findings establish a framework for genomic monitoring and surveillance that can serve as the basis for precision-based containment strategies to combat the spread and transmission of ARGs.

Beyond ARG-harboring plasmid transfer, Wang et al. demonstrated the evolution of resistance by chromosomal integration mechanisms through chromosomal dif sites and associated recombination modules in *Acinetobacter* species. Importantly, the study unravels novel chromosomal mobilization systems that play a critical role in the acquisition and long-term maintenance of ARGs within bacterial species. Therefore, understanding non-plasmid mobilization resistance mechanisms and transmission processes is essential for predicting the evolution of resistance and identifying novel targets for mobilome manipulation.

Another highlight of this Research Topic is an important finding from Jia et al., who reported the emergence of a tigecycline-resistant *Raoultella ornithinolytica* isolate from swine wastewater harboring a highly stable *tet(X4)* encoding plasmid. Detection of conjugative, cross-species transferability and a stable tigecycline-resistant plasmid from pig farm wastewater is of great concern, since tigecycline remains a critical antibiotic for treating multidrug-resistant infections. These observations reinforce an urgent need for integrated One Health surveillance programs to track ARGs across environmental niches before they are transmitted to clinically relevant pathogens.

Besides original research articles with a focus on the spread and dissemination of resistance, the review by López et al. described an evolutionary perspective on fitness costs and persistence of plasmid-mediated cephalosporin resistance in *Escherichia coli*. The authors highlighted the role of fitness, adaptation, and persistence in the stable maintenance of plasmids. Furthermore, the review emphasizes the need to understand plasmid biology and host ecology that shape resistance emergence and persistence within diverse host populations.

In another review, Wang and Liang described an intervention strategy by exploring natural product-based plasmid curing agents. Interestingly, they illustrated plasmid eradication through genetic disarmament, such as disruption or inhibition of replication initiation, partitioning, conjugation, and quorum sensing pathways, rather than bacterial eradication. Furthermore, the study highlighted that the integration of CRISPR technologies, nanotechnology, and artificial intelligence-guided drug discovery should have high potential for developing into next-generation mobilome-centered therapeutics capable of limiting the spread of antimicrobial resistance.

Collectively, the research and review articles in this Research Topic emphasize that mitigation of AMR requires multipronged approaches from pathogen-centered to mobilome-centered intervention strategies. The studies reveal complex MGE networks that govern resistance across the One Health interface. Therefore, focused surveillance efforts and methods to monitor the genetic context of MGEs (plasmids, transposons, integrons, and chromosome-associated mobile elements such as ICE) are required.

Looking forward, the emerging areas of mobilome and microbiome engineering provide an attractive opportunity to develop next-generation antimicrobial strategies. CRISPR-based antimicrobials, MGE removal systems, engineered bacteriophages, and synthetic microbial consortia have considerable promise for selectively targeting resistance determinants without disturbance to resident microbiota. These strategies require a deeper understanding of MGE biology, mechanisms, delivery systems, risk, and regulatory frameworks.

In conclusion, the articles presented in this Research Topic highlight the importance of emerging mobilome research in combating the global problem of AMR. The contributions of this Research Topic will provide a valuable foundation for developing innovative strategies to combat the spread of AMR.
